# Clinical Characteristics of Posner-Schlossman Syndrome Patients in China

**DOI:** 10.1155/2023/4110344

**Published:** 2023-01-20

**Authors:** Tingting Gao, Sijia Song, Xianghan Ke, Shushan Li, Di Zhang, Xuhao Chen, Chun Zhang

**Affiliations:** ^1^Department of Ophthalmology, Beijing Key Laboratory of Restoration of Damaged Ocular Nerve, Peking University Third Hospital, 49 North Garden Road, Haidian District, Beijing 100191, China; ^2^Department of Ophthalmology, Beijing Chaoyang Hospital, Capital Medical University, Beijing, China

## Abstract

**Background:**

To explore the clinical characteristics of Posner-Schlossman syndrome (PSS) patients in China, especially the risk factors associated with the dynamic changes of corneal endothelial cell (CEC) density and retinal nerve fiber layer (RNFL) thickness during the course of the disease.

**Methods:**

In this retrospective cohort study, patients consequently suffering from PSS were recruited. Patients data including gender, age of onset, frequency of attack, and disease duration. We performed detailed ophthalmology examinations including intraocular pressure (IOP), best-corrected visual acuity (BCVA), slit-lamp examination, optical coherence tomography (OCT), assessing RNFL thickness, and determined CEC density in the outpatient department. The unaffected eyes served as control.

**Results:**

We recruited 121 patients (eyes, *n* = 125), including 69 (57.0%) males and 52 (43.0%) females with Posner-Schlossman syndrome. The age of first-onset was 33.81 ± 13.63 years old. The majority of these patients were aged 20-50 years (80.2%). The peak IOP was 47.67 ± 13.31 mmHg in the affected eyes. The frequency of PSS attack was 2.7 ± 3.7 times per year. The disease duration was 9.5 ± 10.4 years. Among all patients, there were 27 (22.31%) patients with a history of digestive disorders, 25 (20.66%) patients with a history of allergies, and 24 (19.83%) patients with a history of infectious disease. In the affected eyes, CEC density (2532.94 ± 490.83/mm^2^ vs. 2777.13 ± 356.87/mm^2^, *p* < 0.001), the RNFL thickness of four quadrants (superior 105.46 ± 29.86 *μ*m vs. 121.33 ± 17.30 *μ*m, *p* < 0.001; temporal 74.58 ± 22.21 *μ*m vs. 81.94 ± 18.20 *μ*m, *p* = 0.009; inferior 110.50 ± 33.42 *μ*m vs. 128.29 ± 14.39 *μ*m, *p* < 0.001; nasal 54.85 ± 14.48 *μ*m vs. 63.49 ± 15.40 *μ*m, *p* < 0.001) and central papillary (87.11 ± 21.18 *μ*m vs. 99.18 ± 7.97 *μ*m, *p* < 0.001) were significantly reduced compared to the fellow eyes. The disease duration and recurrent frequency were significantly associated with CEC density (*p* < 0.001 and *p* = 0.029) and the disease duration was significantly associated with RNFL thickness (*p* = 0.002).

**Conclusions:**

In this study, CEC loss and RNFL thinning were present in the affected eyes. Our results indicated that disease duration and recurrent frequency played an important role in the progression of PSS.

## 1. Introduction

Posner-Schlossman syndrome (PSS), also known as a glaucomatocyclitic crisis, is a rare ocular disease with recurrent attacks of anterior nongranulomatous uveitis, often accompanied by elevated intraocular pressure (IOP) and keratic precipitates (KP) [1]. PSS was first reported by Posner and Schlossman in 1948, and the initial report on PSS suggested that the attacks were unilateral and did not cause permanent damage to the eyes [2]. In the recent two decades, PSS was found to be challenging to cure and also could cause irreversible visual impairment in some patients [3]. Treatments were directed towards controlling the inflammation and related IOP elevation [4]. Even in some cases, surgical management like trabeculectomy was used to treat PSS to control the elevated IOP [5]. Moreover, the dynamic characteristics of pathophysiology in PSS are still not clear. Therefore, it is crucial to recognize the characteristics of PSS to reduce the risk of recurrence and visual damage. In this study, we explore the clinical characteristics of PSS, especially the changes of corneal endothelial cell (CEC) density and retinal nerve fiber layer (RNFL) thickness during the course of the disease and the risk factors associated with the dynamic characteristics.

## 2. Methods

### 2.1. Patients

We recruited PSS patients in the Ophthalmology Department, Peking University Third Hospital from 2014 to 2019. The diagnosis of PSS was based on the definition including following features: (a) recurrent mild iridocyclitis, (b) nonpigmented KP on the central and inferior corneal endothelial surface, (c) cell and flare in the anterior chamber, (d) elevated IOP, (e) no iris posterior synechiae or peripheral anterior synechiae and posterior inflammatory, and (f) a relatively short attack duration [6, 7]. Patients data including IOP and BCVA during the attack, also gender, nationality, age of onset, height, weight, frequency of attack, disease duration, family history, and medical history. The disease duration was calculated based on the data of the last follow-up.

The anterior eye segment of the PSS patients was imaged. The corneal endothelial morphology and numbers were determined by Topcon 2000™ (Topcon, Japan). Peripapillary spectral-domain optical coherence tomography (SD-OCT) was detected by Heidelberg Engineering (Heidelberg, Germany), and the thickness of superior (including superior-temporal and superior-nasal), temporal, inferior (including inferior-temporal and inferior-nasal), nasal peripapillary RNFL of 3.5 mm-diameter subfield, and the central papillary RNFL were recorded. The mean thickness of RNFL was calculated by the average of superior, temporal, inferior, nasal peripapillary RNFL, and central papillary RNFL data. Eyes were divided into the affected group and the unaffected group according to whether they were attacked or not.

Patients were given informed consent to the study. The study protocol was approved by the Ethical Committee of Peking University Third Hospital and was in accordance with the Declaration of Helsinki.

### 2.2. Statistical Analysis

All analyses were performed with SPSS (version 20.0; IBM), and a paired-samples *t*-test was used to compare the differences between the affected group and the unaffected group. Multiple linear regression analysis or one-way ANOVA was performed to test various factors including age of onset, frequency of attack, disease duration, and IOP correlated with CEC density or RNFL thickness. *p* value < 0.05 was considered statistically significant.

## 3. Results

### 3.1. Patient Characteristics

A total of 121 patients (*n* = 125 eyes), including 69 male (57.02%) and 52 female (42.98%) with PSS from Peking University Third Hospital were collected. Among the 121 patients, 119 were Han nationality, one Hui nationality, and one Zhuang nationality. The BMI (body mass index) of male was 24.57 ± 3.00, and the BMI of female was 23.61 ± 3.16. The percentage of patients between 20 to 50 years old was 80.2%. While the percentage of patients younger than 20 years old was 7.4% and older than 50 was 12.4%, respectively. We summarized the patients with PSS by age and gender in Table 1. Among the patients, 117 (96.69%) patients were affected unilateral eye, and 4 (3.31%) were binocular. There was not any laterality (right/left) preference. One or more white mutton-fat precipitates were found on the corneal endothelium in the affected group. The mean logMAR BCVA was 0.17 ± 0.14 in the affected eyes and 0.10 ± 0.23 in the unaffected eyes. There was no significant difference in BCVA between the affected and unaffected eyes (*p* = 0.239). The mean IOP was 47.67 ± 13.31 mmHg (ranged from 20 to 75) in the affected group during an acute attack and 17.10 ± 8.20 in the unaffected group. The IOP of affected eyes was significantly higher than the unaffected eyes (*p* = 0.008). The mean age of patients was 33.81 ± 13.63 years old (ranged from 13 to 70). The recurrent frequency of PSS attack was 2.7 ± 3.7 times per year. The disease duration was 9.5 ± 10.4 years. The characteristics of patients with PSS are listed in Table 2.

Except one patient's mother has PSS, other patients have no family history. Among all patient, there were 27 (22.31%) patients with a history of digestive disorders, 25 (20.66%) patients with a history of allergies, 24 (19.83%) patients with a history of infectious disease, 13 (10.74%) patients with a history of cardiovascular and cerebrovascular disease, and 9 (7.44%) patients had a history of endocrine disease. Among the patients with a history of allergies, 12 patients had a history of allergic rhinitis, one patient had a history of allergic rhinitis and asthma, 5 patients had a history of allergic dermatitis, 2 patients had a history of drug allergy, 2 patients had a history of food allergy, one patient had a history of pollen allergy, one patient had a history of measles, and one patient had a history of pemphigoid. Among the patients with a history of infectious disease, 15 patients were affected with varicella-herpes zoster virus, 3 patients affected with hepatitis B virus, 3 patients affected with herpes simplex virus, 2 patients affected with rubella virus, and one patient with a history of viral meningitis. In addition, there was 1 patient with a history of lymphoma, 1 with breast cancer, 1 with pulmonary sarcoidosis, 1 with renal insufficiency, and 1 with POEMS (peripheral neuropathy, organomegaly, endocrinopathy, monoclonal plasma-cells proliferative disorder and skin changes) syndrome. The disease history of patients with PSS is listed in Table 3. Eighty-eight patients (72.7%) believed that the recurrence of PSS was related to fatigue, stress, mental stress, or staying up late.

### 3.2. CEC Data Analysis

**Figure 1:**
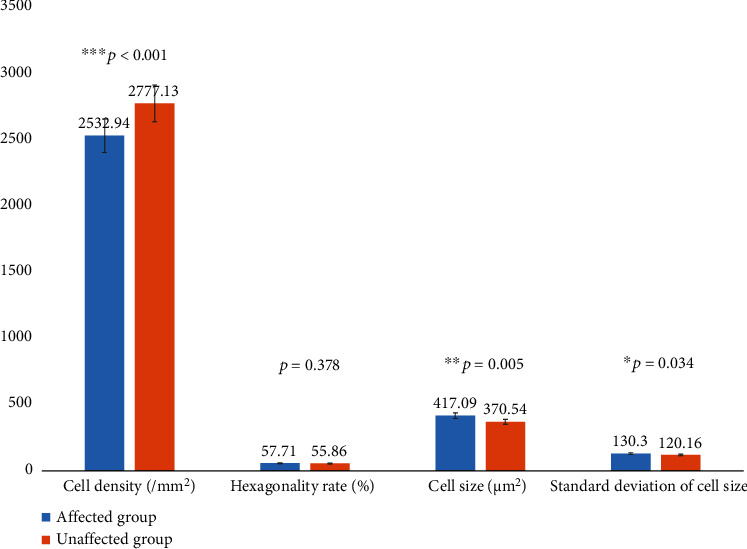
Corneal endothelial cell analysis.

CEC analysis is summarized in Table 4 and Figure 1.

### 3.3. Retinal Nerve Fiber Layer Data Analysis

**Figure 2:**
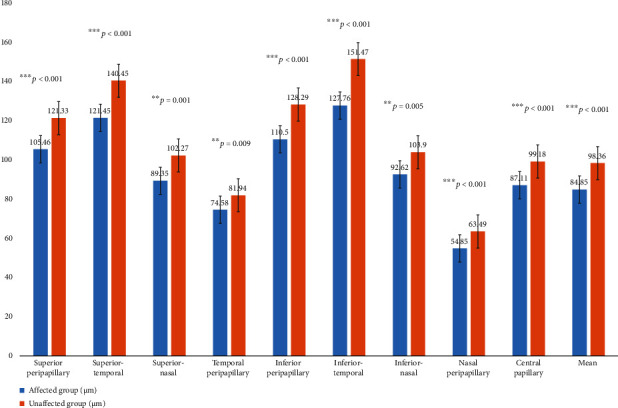
Retinal nerve fiber layer thickness.

The detailed RNFL analysis between the affected group and the unaffected group are enlisted in Table 5 and Figure 2. None of the OCT was measured during the active inflammation period.

### 3.4. Multiple Linear Regression Analysis between Various Factors and CEC Density

Multiple linear regression analysis was performed between various factors including age of onset, frequency of attack, disease duration, and IOP and CEC density (Table 6). There was a significant interaction between the disease duration and CEC density (*p* < 0.001). The recurrent frequency was also significantly associated with CEC density (*p* = 0.029). There was no statistical interaction between the max IOP or age of onset and CEC density (*p* = 0.929 and *p* = 0.479).

### 3.5. ANOVA between Factors and RNFL Thickness

One-way ANOVA was performed between RNFL thickness and factors such as age of onset, frequency of attack, disease duration, and IOP. The disease duration was significantly associated with RNFL thickness (*p* = 0.002). There were no statistical interaction between the max IOP, recurrent frequency, or age of onset and RNFL thickness (*p* = 0.609, *p* = 0.121, and *p* = 0.194).

## 4. Discussion

Posner-Schlossman syndrome (PSS), also known as a glaucomatocyclitic crisis, is a well-known rare ocular disease [4]. The information in our study provides important insights to understand the characteristics of PSS. Our PSS cohort included a majority of Han nationality and a minority of other nationality. A Chinese retrospective study showed that the mean annual incidence of PSS was 3.91 per 100,000 population, and the incidence of PSS was significantly higher in men than women [8], which was similar to our observation. Underlying mechanisms of the privilege distribution in male cases are still waiting to be elucidated. It was referred that the disease was related to the balance-breaking of the immune status [9, 10], and the alternative complement pathway might play an important role in the pathogenesis of PSS [11]. Maintaining a properly functioning adaptive immune system required a proper balance between stimulatory and inhibitory signals [12, 13]. Gender differences in inflammation susceptibility have been found in previous studies [14–16]. It was reported that most patients with PSS were unilateral [17]. Hess et al. reported bilateral simultaneous presentation of PSS [18]. The previous studies were consistent with our finding. Similarly, it is well established that PSS typically affects adults between the ages of 20 and 50 years. PSS in patients aged >70 years and < 20 was considered a rare condition [19, 20].

In this study, one patient developed cataract secondary to long-term steroid use. Therefore, patients who use steroid therapy for a long time should be alert to the occurrence of cataracts. Previously, it was reported that the PSS has a good prognosis despite repeated attacks [21]. Thus, less attention was paid to PSS before. In the recent two decades, irreversible visual impairment was reported in more and more PSS patients.

Our study showed that the CEC density was significantly lower, and the cell size was larger in the eyes affected by PSS. The information also provides evidence of the CEC damage in PSS. One or more white mutton-fat keratic precipitates were found in the corneal endothelium layer in the affected eyes. The cell density of corneal endothelium was significantly lower, while the average cell size of the corneal endothelium was significantly larger in the affected eyes in contrast with the unaffected. The mean value of the standard deviation of cell size was larger in the affected eyes than the unaffected.

The data on the corneal endothelium cells indicated that PSS might decrease the CEC density and the size of the left cells extended. Eventually, there was a worse coherence of cell size in the affected eyes than the unaffected. It was reported that morphological changes of endothelium associated with cell loss occurred in condition of anterior uveitis [22]. There was no effective treatment to recover CEC, so it was important to detect the reason why CEC loss. Previous study showed that the aqueous cytokine may have triggered corneal endothelial cell loss [23]. In this study, we have collected the clinical data of the PSS patients and analyzed the impact factors on CEC density. We found that the long-lasting disease duration and frequent recurrent promoted CEC loss.

Our results showed that the RNFL thickness of all quadrants and central papillary were significantly thinner in the affected eyes than the unaffected eyes. The data also provide evidence of the irreversible impairment in RNFL, which would help to elucidate the pathological change in PSS. A retrospective case series study reported that the global RNFL thickness in eyes affected by PSS was significantly reduced in the follow-up period of 32.8 ± 28.3 months [24]. In previous study, Darchuk et al. reported a PSS case with optic disc damage and visual field loss [25]. Kim et al. also described a pale optic disc and a superior paracentral visual field defect in a 32-year-old man of PSS at his fifth attack [26]. We have also analyzed the impact factors on the dynamic changes of RNFL thickness. Our data showed that long-lasting disease duration caused RNFL thickness thinning. Previous studies showed that the progression of RNFL was associated with frequent attacks of high IOP in young adult patients with PSS [27]. More attention should be given to the retinal nerve fiber damage and elevated IOP in PSS patients.

Moreover, we have collected the family history and systematic history of the PSS patients in this study. Digestive disorders, allergies, and infectious disease were all accounted near one fifth in the PSS patients. And only one patient had family history. Approximately three quarters believed that the recurrence of PSS was related to fatigue, stress, mental stress, or staying up late. The exact etiology of PSS is not clear. The data collected above may provide the risk factors of the disease partially.

Previous study also implied that PSS might be related to vascular endothelial dysfunction [28], inflammatory cytokines [10, 29], allergy [21], genetic susceptibility [30, 31], and viral infection [32]. Many previous studies have reported cytomegalovirus in the aqueous humor or serum [13] and ganciclovir eye drop effective for PSS patients [33, 34].

Our study had some inevitable limitations due to the retrospective design. The diagnosis was based on clinical features of the patients. A virological method including CMV detection will be applied in further studies. Meanwhile, prospective design on features of PSS is suggested to further recognize the features of PSS and explore the pattern of RNFL defects in PSS.

In conclusion, our study indicated that the CEC loss was present in the affected eyes, meanwhile the size of CEC was significantly larger, and a worsen coherence in the eyes was affected with PSS. RNFL analysis suggested that peripapillary in all quadrants, central papillary, and mean RNFL thickness were thinner in the affected eyes. We found that the long-lasting disease duration and frequent recurrent promoted CEC loss, and long-lasting disease duration caused RNFL thickness thinning.

## Figures and Tables

**Table 1:** The summary of patients with PSS by age and gender.

Age (year)		Male		Female		Total
		*n* (%)		*n* (%)		*n* (%)
<20	3	2.5%	6	5.0%	9	7.4%
20-50	56	46.3%	41	33.9%	97	80.2%
>50	10	8.3%	5	4.1%	15	12.4%
Total	69	57.0%	52	43.0%	121	100%

**Table 2:** The characteristics of patients with PSS.

	Total	Percentage	*p* value
Affected eyes			
Unilateral eye	117	96.69%	
Bilateral eyes	4	3.31%	
BCVA (logMAR, mean ± SD)			0.239
Affected eyes	0.17 ± 0.14		
Unaffected eyes	0.10 ± 0.23		
IOP (mmHg, mean ± SD)			0.008
Affected eyes	47.67 ± 13.31		
Unaffected eyes	17.10 ± 8.20		
Age (year, mean ± SD)	33.81 ± 13.63		
Recurrent frequency (times per year, mean ± SD)	2.7 ± 3.7		
Disease duration (year, mean ± SD)	9.5 ± 10.4		
Nationality			
Han nationality	119	98.34%	
Other nationality	2	1.66%	

**Table 3:** The disease history of patients with PSS.

	Case number	Percentage
PSS family history	1	0.83%
Digestive disorders	27	22.31%
Allergies	25	20.66%
Infectious disease	24	19.83%
Cardiovascular and cerebrovascular disease	13	10.74%
Endocrine disease	9	7.44%
Lymphoma	1	0.83%
Breast cancer	1	0.83%
Pulmonary sarcoidosis	1	0.83%
Renal insufficiency and 1 with	1	0.83%
POEMS syndrome	1	0.83%

**Table 4:** CEC analysis between the affected group and the unaffected group.

	Affected group	Unaffected group	*p* value
Cell density (/mm^2^)	2532.94 ± 490.83	2777.13 ± 356.87	<0.001
Hexagonality rate (%)	57.71 ± 10.11	55.86 ± 10.86	0.378
Cell size (*μ*m^2^)	417.09 ± 127.99	370.54 ± 58.77	0.005
Standard deviation of cell size	130.30 ± 47.36	120.16 ± 31.55	0.034

**Table 5:** RNFL analysis between the affected group and the unaffected group.

RNFL thickness	Affected group (*μ*m)	Unaffected group (*μ*m)	*p* value
Superior peripapillary	105.46 ± 29.86	121.33 ± 17.30	<0.001
Superior-temporal	121.45 ± 36.42	140.45 ± 17.59	<0.001
Superior-nasal	89.35 ± 26.92	102.27 ± 24.30	0.001
Temporal peripapillary	74.58 ± 22.21	81.94 ± 18.20	0.009
Inferior peripapillary	110.50 ± 33.42	128.29 ± 14.39	<0.001
Inferior-temporal	127.76 ± 43.02	151.47 ± 14.68	<0.001
Inferior-nasal	92.62 ± 28.44	103.90 ± 26.10	0.005
Nasal peripapillary	54.85 ± 14.48	63.49 ± 15.40	<0.001
Central papillary	87.11 ± 21.18	99.18 ± 7.97	<0.001
Mean	84.85 ± 18.86	98.36 ± 6.43	<0.001

**Table 6:** Multivariate regression analysis of CEC density and various factors.

Variable	Estimate	Standard error	B0	*p* value
Max IOP	0.616	6.829	0.019	0.929
Disease duration	-29.619	6.419	-0.883	< 0.001
Recurrent frequency	88.246	37.395	0.422	0.029
Age	-5.166	7.157	-0.141	0.479

## Data Availability

The data used to support the findings of this study are included within the article.
